# Central Nervous System Alterations in Individuals with Long COVID: A Systematic Review

**DOI:** 10.1590/0037-8682-0519-2026

**Published:** 2026-10-16

**Authors:** Janaina Ferreira de Moraes, Paulo Henrique Leite Souza, Michele Lacerda de Andrade, Raphael Mendes Ritti Dias, Fernanda Ishida Corrêa

**Affiliations:** 1 Universidade Nove de Julho (UNINOVE), Programa de Mestrado e Doutorado em Ciências da Reabilitação, São Paulo, SP, Brasil.

**Keywords:** Long COVID, Neurophysiology, Fatigue, Cognitive dysfunction, Rehabilitation

## Abstract

Long COVID (post-COVID-19 condition) has been associated with persistent neurological and cognitive symptoms, suggesting possible central nervous system involvement beyond the acute phase of severe acute respiratory syndrome coronavirus 2 infection. A systematic review of articles published up to April 2026 was conducted following the PRISMA guidelines, and observational studies evaluating neurophysiological, structural, and functional alterations in individuals with long COVID were analyzed. Forty-two studies with substantial variability in design and methods were included; thus, the findings were synthesized narratively. The evidence indicates heterogeneous yet recurrent alterations in the central nervous system of individuals with lonf COVID, including impaired cortical excitability, abnormal cerebral oscillatory activity across multiple frequency bands, changes in functional connectivity, and structural abnormalities in neural networks. Some studies also reported imbalances in excitatory-inhibitory neurotransmission and biomarkers suggestive of neuroinflammation or axonal injury. These neurophysiological and structural alterations were commonly associated with persistent fatigue, cognitive dysfunction, and brain fog. However, the available evidence is limited by important methodological issues, including substantial heterogeneity in study populations, assessment techniques, and reported outcomes; the observational design also limited the strength of the conclusions. Taken together, the current evidence suggests a persistent and multifactorial central nervous system dysfunction in long COVID. However, definitive conclusions remain limited, and well-designed prospective longitudinal studies using standardized methods are needed to clarify the mechanisms, duration, and clinical relevance of these alterations.

## INTRODUCTION

The term “long COVID”, also known as post-COVID-19 condition, refers to the signs and symptoms that persist for more than 12 weeks after infection with severe acute respiratory syndrome coronavirus 2 (SARS-CoV-2), with no other identified cause^1^. More than 200 symptoms have been reported, the most common of which are fatigue, dyspnea, headache, attention deficit, and alopecia, in addition to manifestations such as myalgia, radiological changes, and cardiac complications^2^ ^-^ ^4^. 

The neurotropic potential of SARS-CoV-2 is notable, as the virus is capable of directly invading the central nervous system (CNS) and causing neurological manifestations, especially in severe cases. Such manifestations include delirium, brain inflammation, stroke, neuropathies, encephalopathy, encephalitis, seizures, Guillain-Barré syndrome, and sensory dysfunctions, such as anosmia and dysgeusia. Furthermore, the pandemic has intensified psychological impacts, such as anxiety, mood disorders, and post-traumatic stress, often associated with long COVID^5^ ^-^ ^7^.

Given the challenges posed by long COVID, there is a need to expand knowledge regarding neurological impairments, which are often underestimated or unrecognized as part of the infectious process. Failure to consider these manifestations can negatively impact engagement and adherence to rehabilitation programs and eventually lead to persistent debilitating clinical consequences. Clarifying the neurophysiological basis of these symptoms is a crucial step toward developing appropriate, targeted, patient-centered rehabilitation interventions.

The present review seeks to fill this gap by synthesizing evidence on CNS changes in long COVID and discussing the direct implications of such changes to improve the rehabilitation process. Specifically, the study aimed to a) identify the main neurophysiological changes observed in the CNS of individuals with long COVID and their respective clinical symptoms and b) analyze the duration of these manifestations. The goal is to provide insights into the possible clinical sequelae of the disease and formulate more effective future rehabilitation strategies. 

Beyond characterizing neurophysiological alterations and their duration, this review critically examines their clinical correlates, discusses the methodological heterogeneity that limits the current evidence, and explores the practical implications for clinical screening and the design of individualized, multidisciplinary rehabilitation strategies.

## METHODS

### Study Design and Registration

This systematic review was conducted in accordance with the Preferred Reporting Items for Systematic Reviews and Meta-Analyses (PRISMA 2020) guidelines^8^. The study protocol was prospectively registered in the International Prospective Register of Systematic Reviews (PROSPERO: CRD42024554862).

The review question was structured using the PEO framework (Population, Exposure, Outcome), which is considered appropriate for systematic reviews of observational studies. The components were defined as follows: individuals diagnosed with Long COVID as the Population, previous SARS-CoV-2 infection associated with persistent symptoms as the Exposure; and neurophysiological alterations in the CNS identified through validated neurophysiological assessment methods as the Outcome.

### Eligibility Criteria

Studies were considered eligible if they met the following criteria: (a) observational design (including cross-sectional, cohort, or longitudinal studies); (b) population composed of individuals of both sexes diagnosed with long COVID; (c) investigation of neurophysiological or functional changes in the CNS associated with post-COVID conditions; and (d) inclusion of human participants. No restrictions were applied regarding language or publication date. 

Studies were excluded if they were (a) narrative reviews, qualitative studies, or case reports or (b) conducted in participants with a history of neurological disorders prior to COVID-19 diagnosis, considering the potential confounding effect on the neurophysiological outcomes.

### Search Strategy

The search strategy was developed in accordance with current recommendations for systematic reviews, using a combination of controlled vocabulary (MeSH, Emtree, and DeCS) and free-text terms related to long COVID and neurophysiological changes in the CNS. 

Searches were conducted in PubMed/MEDLINE, Embase, LILACS, SciELO, and the Cochrane Library from database inception to the date of the final search. Only studies involving human participants were considered.

The search strategy was designed to maximize sensitivity for identifying relevant observational studies and did not use predefined database study-design filters (e.g., *Systematic Review* or *Meta-Analysis*), thereby avoiding the inadvertent exclusion of eligible primary studies. Although terms related to study designs (e.g., *cross-sectional*, *cohort*, *longitudinal*, and *observational*) were included as free-text keywords to improve search sensitivity, they were not applied as methodological filters to restrict the search results.

The search terms included variations of “Long COVID,” “Post-Acute COVID-19 Syndrome,” “Post-COVID-19 Condition,” and “Post-Acute Sequelae of SARS-CoV-2 Infection,” combined with terms related to CNS function and neurophysiological assessment (e.g., electroencephalography, transcranial magnetic stimulation, neural activity, and neurotransmitters). 

To ensure methodological rigor, all searches were independently conducted by two reviewers. In addition, the reference lists of all included studies were screened to identify potentially eligible articles not captured in the electronic search. A complementary manual search was also performed to broaden the bibliographic coverage and minimize the risk of publication bias.

The complete search strategies for each database, including the full Boolean expressions and controlled vocabulary terms, are provided in Supplementary Table S1 to ensure transparency and reproducibility.

### Study Selection

After the removal of duplicates, the studies were screened in two stages by two independent reviewers: (1) title and abstract screening and (2) full-text assessment according to the predefined eligibility criteria. Disagreements were resolved by consensus or by consultation with a third reviewer.

The study selection process, including the identification of studies through databases and other methods as well as detailed reasons for exclusion at the full-text stage, is presented in a PRISMA 2020 flow diagram.

### Data Extraction and Outcomes

Data extraction was performed independently by two reviewers using a standardized form.

The following information was collected: authorship, year of publication, country, study design, sample characteristics (number of participants, age, and sex), clinical features, duration of symptoms, neurophysiological assessment methods, and main findings related to CNS alterations. To address the heterogeneity of neurophysiological approaches across studies, the outcomes were extracted in a structured but non-hierarchical manner.

The column “Neurophysiological assessment modalities and outcome measures” was used to systematically capture both the neurophysiological techniques employed (e.g., magnetic resonance imaging [MRI], electroencephalography [EEG], transcranial magnetic stimulation [TMS], positron emission tomography [PET], functional near-infrared spectroscopy [fNIRS], and multimodal approaches) and the corresponding quantitative or clinical variables reported in each study.

No formal categorization or grouping of outcomes was applied at the extraction stage; instead, all variables were recorded as reported in the original studies. Discrepancies during data extraction were resolved by consensus or by consultation with a third reviewer.

### Risk of Bias Assessment

The methodological quality of the included studies was assessed using the Joanna Briggs Institute (JBI) critical appraisal tools selected according to the study design. Cross-sectional studies were evaluated using the JBI Critical Appraisal Checklist for Analytical Cross-Sectional Studies^9^, whereas cohort and longitudinal studies were assessed using the JBI Critical Appraisal Checklist for Cohort Studies^10^. Two independent reviewers performed the assessments, and disagreements were resolved through consensus.

### Protocol Deviations

Deviations from the original protocol were necessary to improve methodological rigor and ensure alignment with the study objectives. These included refinement of the search strategy, removal of inappropriate study-type filters, expansion of eligibility criteria to include a broader range of observational designs, and the adoption of a more appropriate tool for risk of bias assessment in accordance with the study design.

Additionally, although a meta-analysis was initially planned, it was not conducted owing to substantial heterogeneity across studies in terms of design, neurophysiological assessment methods, and outcome reporting. Therefore, a structured narrative synthesis was performed.

All deviations from the protocol are transparently reported and justified in the present manuscript.

RESULTS

The initial search identified 597 records. After the removal of 42 duplicates, 555 records were screened by title and abstract. Of these, 499 were excluded for the following reasons: systematic reviews, qualitative studies, case reports, protocol articles, studies on other pathologies, abstracts of clinical trials, conference/congress abstracts, use of other measurement objectives, and clinical trials. Fifty-six full-text articles were assessed for eligibility. Among these, 14 articles were excluded: other pathologies, clinical trials without relevant CNS outcomes, and use of other measurement objectives. Ultimately, 42 studies met all the inclusion criteria and were included in this review (Supplementary Figure S1).

### General Characteristics of Included Studies

Notable, 42 studies were included (Table 1), comprising 39 adult and 3 pediatric cohorts. Most studies (88.1%) included healthy control groups. Cross-sectional and longitudinal observational designs predominated. The assessment modalities included structural and functional MRI, diffusion tensor imaging (DTI), EEG, TMS, PET, single-photon emission computed tomography, fNIRS, and blood biomarkers.

**TABLE 1:** Characteristics and main findings of studies evaluating central nervous system alterations in long COVID.

Author/year	Country	Study design	Study objective	Sample characteristic	Neurophysiological technique	Results/Conclusion
Douaud et al.^11^	United Kingdom	Observational longitudinal cohort	Investigating the structural changes in the brain	EG: General population (COVID-19 diagnosis): n=401; 58.9±7.0 years; 172M/229F; follow-up: 141±79 days (35-407); 15 hospitalized CG: General population (no infection): n=384; 60.2±7.4 years; 164M/220F	Technique: Structural MRI, DTI Measurements: Cortical thickness, brain volume, MD; OD; ISOVF	The EG showed a greater reduction in cortical thickness in the lateral orbitofrontal cortex and left parahippocampal gyrus; increased markers of tissue damage (MD, ISOVF, OD) in the regions connected to the olfactory cortex; reduced global brain volume and increased CSF volume; worsened cognitive performance (TMT A/B) compared with the CG. Effects were independent of hospitalization.
Hartung et al.^12^	Germany	Cross-sectional	Assess structural brain changes and their associations with fatigue and cognitive performance.	EG (COVID-19 group): n = 57; 53 ± 13 years; 36M/21F; 3 months post-discharge; 45 moderate/12 severe. CG (healthy controls): n = 57; 50.5 years; 31M/26F; sex- and age-matched.	Technique: 3T structural MRI. Measures: Cortical thickness; subcortical volumes.	The EG showed reduced cortical thickness in the temporal regions (parahippocampal gyri, temporal poles) and right frontal cortex; reduced bilateral hippocampal volume; greater disease severity was associated with lower cortical thickness in olfactory-related regions; fatigue was associated with reduced cortical thickness (anterior/posterior cingulate and temporal regions) compared with the CG; no significant association with cognitive performance (MoCA); severe cases showed reduced thalamic volume.
Esposito et al.^46^	Italy	Observational longitudinal cohort	Assess changes in the olfactory connectivity after COVID-19 and its evolution over a 1-year period	EG (COVID-19 group): n = 18; 38.7 ± 8.1 years; 10M/8F; baseline assessment ~52 days post-infection; follow-up ~385 days. CG (healthy controls): n = 10; 33.1 ± 3.6 years; 5M/5F.	Structural MRI with structural connectivity analysis. Measure: Connectivity between the primary olfactory cortex and mOFC.	Increased olfactory structural connectivity (mOFC) in the early post-COVID phase; normalization after 1 year; alterations independent of olfactory function recovery; suggesting a reversible nature of connectivity changes.
Samanci et al.^50^	Turkey	Cross-sectional	Investigate structural, functional, and cognitive changes after mild COVID-19, with and without brain fog	EG1 (no brain fog): n = 26; 30 years; 10M/16F; ~348 days post-COVID. EG2 (with brain fog): n = 25; 35 years; 8M/17F; ~375 days post-COVID. CG (healthy controls): n = 24; 30 years; 14M/11F.	Structural MRI + resting-state fMRI. Measures: Gray matter; functional connectivity (DMN, DAN, visual networks).	Both EGs showed reduced gray matter in the temporal and orbitofrontal regions; additional reductions in subcortical nuclei (caudate, putamen, amygdala) in individuals with brain fog; altered functional connectivity with increased connectivity in the default mode and visual networks and decreased connectivity in the dorsal attention network; worse cognitive performance in individuals with brain fog; suggesting more pronounced structural and functional alterations in the presence of persistent cognitive symptoms.
Seo et al.^13^	South Korea	Cross-sectional study with an independent validation sample.	Investigate the structural and neurodegenerative changes in individuals with PASC and cognitive impairment.	EG (cognitive PASC): n = 28 (exploration) + 16 (validation); ~64 years; predominantly female; ~13-19 months post-COVID. CG (PASC without significant symptoms): n = 77 (exploration) + 46 (validation); ~56 years; predominantly female.	Technique: Structural MRI + DTI. Measures: Cortical thickness; diffusivity (MD, RD, FA); susceptibility; choroid plexus volume; ALPS index; MD; RD; FA.	Cortical thinning in cingulate, insular, and parahippocampal regions; increased susceptibility (iron deposition) in the cortical and subcortical regions; white matter microstructural alterations (increased MD/RD and reduced FA); increased choroid plexus volume and reduced ALPS index (glymphatic dysfunction); alterations in neurodegenerative and inflammatory biomarkers; findings confirmed in a validation cohort; suggesting a more pronounced neurodegenerative profile in PASC with cognitive impairment.
Tian et al.^32^	China	Observational longitudinal cohort	Investigate structural and perfusion-related brain changes after COVID-19 in individuals without neurological manifestations.	EG1 (mild): n = 13; 58.1 ± 5.7 years; 6M/7F; ~10 months post-discharge. EG2 (severe): n = 21; 62.8 ± 5.4 years; 10M/11F; ~10 months post-discharge. CG (healthy controls): n = 31; 60.6 ± 6.4 years; 18M/13F.	Technique: Structural MRI + ASL (perfusion) + DTI. Measures: Cortical thickness; cerebral blood flow; subcortical volumes; white matter.	Cerebral hypoperfusion in cortical and subcortical regions, more pronounced in severe cases; reduced volume in white matter tracts and subcortical nuclei; alterations also present in mild cases; partial recovery of perfusion over time, with persistence of structural changes; suggesting the coexistence of reversible and persistent alterations after COVID-19.
Scardua-Silva et al.^35^	Brazil	Cross-sectional	Investigate microstructural and cognitive changes after mild COVID-19 in individuals without anxiety or depression	EG (post-mild COVID without hospitalization): n = 97; 39 years; predominantly female; ~79 days post-infection. CG (healthy controls): n = 77; 36 years; predominantly female.	Technique: DTI + resting-state fMRI. Measures: Diffusivity (AD, FA, MD, RD); functional connectivity (DMN)	Compared with the CG, the EG showed increased AD in white matter tracts; no changes in FA, MD, or RD; no differences in DMN functional connectivity; presence of cognitive deficits and fatigue in a subset of patients; suggesting subtle microstructural alterations associated with cognitive dysfunction after mild COVID-19.
Schmidt et al.^23^	Germany	Cross-sectional	Investigate reward processing and cognitive mechanisms associated with fatigue in post-COVID syndrome	EG (post-COVID with chronic fatigue): n = 24; 37.1 ± 7.4 years; 15F/9M; ~17.9 months post-infection. CG (healthy controls): n = 24; 34.6 ± 12.4 years; 10F/14M.	Task-based fMRI (reward task). Measures: Reaction time; brain activation; pupillary response.	Longer reaction time in post-COVID individuals; preserved reward processing; delayed brain activation in the frontal regions; association with signs of neural hypoarousal (pupillary alterations); suggesting that fatigue is related to cerebral hypoarousal rather than deficits in the reward system.
Petersen et al.^19^	Germany	Longitudinal cohort	Examine the association between the olfactory bulb volume and progression of post-COVID olfactory dysfunction.	N = 233 (follow-up), n = 143 (final analysis), 55.79 ± 7.25 years, 44.2% female.	Technique: Structural MRI and quantitative olfactometry. Measures: Olfactory bulb volume; olfactory function.	143 participants completed follow-up (81 losses). Self-reported olfactory dysfunction decreased from 67.1% in the acute phase to 21.0% at baseline and 17.5% at follow-up. Persistent cases showed reduced olfactory bulb volume, which was associated with future olfactometric performance and clinical recovery. No association was found between olfactory bulb volume and neuropsychological performance.
Huang et al.^34^	China	Observational longitudinal cohort	Investigate long-term microstructural changes in white matter after COVID-19 and their association with cognition and inflammation	EG (recovered from COVID-19): n = 17; 53.8 ± 10.2 years; 9M/8F. CG (healthy controls): n = 13; 51.5 ± 12.6 years; 1M/12F.	Technique: DTI and NODDI. Measures: White matter integrity; cognition; inflammatory markers. Diffusion metrics: FA, RD, AD, MD; ODI, Vic (intracellular water fraction), Viso (isotropic fraction). Voxel-wise analysis (TBSS) with FWE correction. Cognitive measures: LM, DSST, DS, WFT.	The EG showed persistent white matter alterations, with increased free water and signs of microstructural damage; evidence of late focal deterioration between 1 and 2 years after infection; worse performance in episodic memory; association between acute inflammatory markers and white matter and cognitive changes; suggesting a prolonged impact of inflammation on brain integrity.
Andriuta et al.^47^	France	Cross-sectional	Characterize neurocognitive deficits and their association with white matter alterations in post-COVID patients with cognitive complaints	EG (cognitive complaints): n = 46 (MRI: n = 36); 50.9 ± 14 years; ~254 days post-infection; predominantly female. CG (healthy controls): n = 1,003; 62 ± 11.3 years; predominantly female.	Technique: Brain MRI. Measures: White matter hyperintensities; cognitive performance.	The EG showed worse global cognitive performance, with a predominance of psychomotor slowing and executive deficits; presence of neurocognitive disorder in a subset of patients; the need for oxygen during the acute phase was associated with worse cognitive performance; white matter hyperintensities were associated with cognitive deficits, especially in the right frontal regions; suggesting a relationship between clinical severity and structural brain changes.
Chen et al.^29^	China	Cross-sectional	Investigate changes in brain activation associated with long COVID during motor and cognitive tasks.	EG (Long COVID): n = 437 (fNIRS subgroup: n = 39); ~28 years; predominantly mild symptoms; 54.2% female with mild symptoms. CG (healthy controls): n = 61 (fNIRS subgroup: n = 10); age- and sex-matched.	Technique: fNIRS. Measures: Brain activation in the motor and cognitive regions; functional performance.	The EG showed reduced activation in frontal and cognitive cortical regions during motor tasks; altered activation of motor areas associated with the degree of functional impairment; greater severity of initial symptoms associated with lower brain activation; suggesting dysfunction in the integration between motor networks.
Churchill et al.^52^	Canada	Cross-sectional	Investigate changes in brain functional dynamics (Hurst exponent) associated with post-COVID-19 syndrome and persistent headache	EG1 (no headache): n = 32; 40.3 ± 12.5 years; 66% female; ~120 days post-infection. EG2 (persistent headache): n = 14; 46.1 ± 10.1 years; 79% female; ~112 days post-infection. EG3 (resolved headache): n = 11; 36.2 ± 10.6 years; 55% female; ~124 days post-infection. CG (no COVID-19): n = 17; 41.5 ± 13.1 years; 59% female.	Technique: fMRI. Measure: Hurst exponent (H); functional activity and connectivity (ALFF, fALFF).	Reduced global and regional Hurst exponent in the persistent headache group (inferior temporal cortex, sensorimotor regions, and insula); the resolved headache group showed higher H values compared with both the persistent headache and no-headache groups; the no-headache group did not differ from the CG; reductions in ALFF, fALFF, and local connectivity in the same regions; findings suggest altered brain functional dynamics associated with persistent headache, with reversibility after symptom resolution.
Klironomos et al.^20^	Sweden	Retrospective consecutive cohort	Describe the frequency and type of neuroradiological findings in hospitalized patients with COVID-19	EG (hospitalized post-COVID-19 with neuroimaging): n = 185; 62 ± 14 years; 138M/47F; ~34 days since symptom onset; 62% mechanical ventilation; 25% mortality. CG: not applicable.	Technique: CT and brain MRI. Measures: Structural findings (hemorrhage, ischemia, white matter abnormalities, contrast enhancement, cranial nerves, olfactory bulbs, perfusion).	High frequency of susceptibility abnormalities on MRI (predominantly in the corpus callosum and white matter); presence of periventricular leukoencephalopathy; meningeal, cranial nerve, and nerve root enhancement in a subset of patients; abnormalities in the olfactory bulbs and optic nerves; the pattern of findings suggests vascular and inflammatory involvement of the nervous system, with possible thrombotic microangiopathy.
Huang et al.^36^	China	Cross-sectional	Investigate long-term white matter alterations and cognitive function after COVID-19	EG (recovered from COVID-19): n = 22; 54.1 ± 9.8 years; 11M/11F; ~1 year post-discharge; ICU subgroup: n = 8; non-ICU: n = 14. CG (healthy controls): n = 21; 49.1 ± 12.4 years; age-, sex-, and education-matched.	Technique: MRI (DTI, DKI, NODDI). Measures: White matter microstructural parameters (FA, RD, AD, MD, MK, ODI, Vic, Viso); cognitive function.	The EG showed reduced intracellular water fraction (Vic) in white matter tracts (corona radiata, corpus callosum, superior longitudinal fasciculus); no significant differences in other DTI, DKI, and NODDI parameters; the ICU subgroup showed lower FA in the corpus callosum compared with the non-ICU group; no differences in cognitive function between groups; Vic was negatively correlated with hospital length of stay and positively correlated with follow-up time; findings suggest subtle white matter microstructural alterations with possible recovery over time.
Kandemirli et al.^16^	Turkey	Prospective cross-sectional	Assess changes in the olfactory bulb and olfactory cleft in patients with persistent post-COVID-19 anosmia	EG (persistent anosmia): n = 23; ~29 years; 14F/9M; 1-4 months post-infection. CG: not applicable.	Technique: CT of the paranasal sinuses and MRI. Measures: Olfactory system structures (olfactory cleft, olfactory bulb, olfactory sulcus, and olfactory cortex).	High frequency of olfactory cleft opacification; reduced olfactory bulb volume in a subset of patients; morphological abnormalities of the olfactory bulb and shallow olfactory sulcus; signal abnormalities in the olfactory bulb and, less frequently, in the olfactory cortex; findings suggest structural impairment of the olfactory system associated with persistent post-COVID-19 anosmia.
Haider et al.^21^	Austria	Cross-sectional	Assess brain morphological abnormalities after mild to moderate COVID-19	EG (recovered from COVID-19): n = 112; 45 ± 15 years; 70F/42M; ~7.6 months post-infection; 30 hospitalized. CG (healthy controls): n = 50; 44 ± 19 years; 28F/22M.	Technique: MRI. Measures: Structural brain findings (lesions, microhemorrhages, perfusion, WMH, perivascular spaces, volumetry).	No differences in white matter hyperintensity burden or perivascular spaces between groups; no abnormalities in diffusion, perfusion, susceptibility, or angiography; no differences in brain volumetry between groups; subgroup analysis of hospitalized and non-hospitalized participants also showed no differences; findings suggest no detectable structural brain alterations after mild to moderate COVID-19.
Hosp et al.^49^	Germany	Cross-sectional	Assess cognitive impairment and its neural correlates in hospitalized COVID-19 patients in the subacute phase	EG (hospitalized COVID-19): n = 29; 65.2 ± 14.4 years; neurological symptoms; assessment ~18-31 days after symptom onset. CG (controls): n = 45 (PET) + n = 35 (validation); age-matched.	Technique: 18FDG-PET + MRI. Measures: Cerebral metabolism; cognitive performance (MoCA).	The EG showed diffuse frontoparietal hypometabolism; a specific metabolic pattern associated with COVID-19; alterations correlated with worse cognitive performance; suggesting neocortical dysfunction in the subacute phase of the disease.
Hosp et al.^44^	Germany	Cross-sectional	Assess cerebral blood flow and its association with fatigue, cognition, and olfaction in the post-COVID condition	EG (post-COVID): n = 55; ~39 years; ~10.5 months post-infection; high prevalence of fatigue. CG (recovered without post-COVID): n = 36; similar age.	Technique: 3T MRI with ASL. Measures: Regional cerebral blood flow; clinical scales (fatigue, cognition, olfaction).	No significant differences in CBF were found between groups after correction; exploratory analyses showed reduced perfusion in occipital regions; lower CBF was associated with greater fatigue, especially physical fatigue; suggesting a relationship between cerebral hypoperfusion and fatigue in the post-COVID condition.
Zingaropoli et al.^30^	Italy	Longitudinal observational	Investigate biomarkers of neuronal injury and immune activation and their association with neurological symptoms in COVID-19	EG (hospitalized COVID-19): n = 144; ~64 years; moderate to severe cases; assessment at admission and after 3 months. CG (healthy controls): n = 53; ~64 years; 23M/30F.	Technique: Biomarker analysis in plasma and CSF (cerebrospinal fluid). Measures: NfL, GFAP, sCD163, and inflammatory markers.	The EG showed elevated levels of neuronal injury and immune activation biomarkers in the acute phase; higher levels were associated with greater disease severity and neurological symptoms; partial reduction after 3 months, but still above control levels; suggesting persistent involvement of inflammation and neuronal damage in COVID-19.
Leitner et al.^43^	Austria	Cross-sectional (case-control)	“Investigate changes in thalamic functional connectivity associated with post-COVID fatigue	EG1 (post-COVID with fatigue): n = 15; 52.4 ± 8.8 years; 12M/3F; mean time since diagnosis: 18.1 ± 10.4 months. EG2 (post-COVID without fatigue): n = 24; 48.5 ± 8.4 years; 21M/3F. CG: not available / not included.	Technique: 3T resting-state functional MRI. Measures: Thalamic functional connectivity; cognitive performance; fatigue.	The fatigue group showed reduced/anticorrelated functional connectivity between the thalamus and motor regions; these alterations were associated with greater fatigue and worse processing speed performance; no relevant structural differences were observed between groups; suggesting a functional (rather than structural) basis for post-COVID fatigue.
Bispo et al.^17^	Brazil	Cross-sectional	Investigate changes in the olfactory bulb volume and structural connectivity in patients with post-COVID olfactory dysfunction	EG (COVID-19 with olfactory dysfunction): n = 38; 36.4 ± 9.5 years; 28F/10M; mild infection. CG (healthy controls): n = 24; 39.3 ± 12.9 years; 16F/8M.	Technique: 3T MRI with DTI. Measures: Olfactory bulb volume; structural connectivity; olfactory performance and cognitive performance (MoCA).	The EG showed reduced olfactory bulb volume; alterations in structural connectivity with lower global efficiency and higher network segregation; increased connectivity in parietal regions; suggesting cerebral structural reorganization associated with post-COVID olfactory dysfunction.
Du et al.^14^	China	Prospective longitudinal	Investigate changes in the gray matter volume over 1 and 2 years after COVID-19 and their association with clinical markers.	EG (post-COVID): n = 22 (1 year), n = 18 (2 years); 54.1 ± 9.8 years (1 year), 53.5 ± 10.0 years (2 years). CG (healthy controls): n = 27; 50.8 ± 11.5 years; age-, sex-, and education-matched.	Technique: 3T MRI (VBM). Measures: Gray matter volume; clinical and cognitive symptoms.	The EG showed a reduced volume in the cerebellar and temporal regions; partial recovery was observed in the frontal and temporal areas over time; persistent volume reduction in the cerebellar and temporal regions after 2 years; alterations were associated with acute-phase inflammatory markers; suggesting a prolonged effect of inflammation on post-COVID brain structure.
Nakase et al.^51^	Japan	Cross-sectional observational	Investigate mechanisms of brain injury in patients with long COVID (“brain fog”) using neuroimaging and blood biomarkers	EG (post-COVID brain fog): n = 33; 38.5 ± 14.8 years; 16F/17M. Mean time since infection: 7.4 ± 7.3 months; some participants showed cognitive impairment (MoCA < 26). CG: no direct control group (comparison with normative database).	Technique: 3T MRI + SPECT + blood biomarkers. Measures: rCBF; white matter lesion volume; cognitive tests; biomarkers (GFAP, NfL, Aβ42/40).	Correlation analysis (adjusted for age and sex): Reduced rCBF in the left occipital cortex was associated with worse cognitive performance; right occipital hyperperfusion and right parietal hypoperfusion were associated with neuropsychiatric symptoms; worse cognitive performance was associated with elevated GFAP levels; better cognitive performance was associated with higher NfL levels; no association between cognition and white matter lesion volume; suggesting perfusion dysfunction and astroglial/axonal involvement in “brain fog.”
Churchill et al.^42^	Canada	Cross-sectional (case-control)	Investigate the subcortical microstructural changes associated with persistent fatigue in post-COVID	EG1 (PACS with fatigue): n = 16; 44.8 ± 10.1 years; 12F/4M. EG2 (PACS without fatigue): n = 32; 39.5 ± 12.8 years; 20F/12M. CG (influenza-like symptoms, SARS-CoV-2 PCR negative): n = 15; 41.4 ± 14.0 years; 9F/6M. EG1 (PACS with fatigue): n = 16; ~44.8 years; ~3.7 months post-infection. EG2 (PACS without fatigue): n = 32; ~39.5 years. CG (non-COVID): n = 15; comparable age.	Technique: 3T MRI. Measures: Texture analysis (Haralick features) in subcortical nuclei and brainstem.	No differences were found between the overall COVID group and CG; patients with fatigue showed reduced energy and increased entropy in the putamen/pallidum and ventral diencephalon; reduced correlation and clustering metrics in the thalamus and diencephalon; alterations were also present compared with controls; the pattern indicates greater tissue heterogeneity and possible diffuse microstructural damage; suggesting an association between persistent fatigue and subtle subcortical alterations detectable through texture analysis.
Chang et al.^22^	United States of America	Cross-sectional	Investigate changes in brain activation during working memory in individuals with post-COVID and neuropsychiatric symptoms.	EG (post-COVID): n = 29; 42.4 ± 12.3 years; 19F/10M; high prevalence of fatigue, cognitive and emotional symptoms. CG (healthy controls): n = 21; 41.5 ± 12.2 years; 12F/9M.	Technique: 3T functional magnetic resonance imaging (fMRI) Measures: BOLD activation during working memory task (N-back); cognitive and motor performance, and neuropsychiatric symptoms	The EG showed preserved cognitive and task performance; greater activation in the superior frontal gyrus during high cognitive load; reduced deactivation of the Default Mode Network (posterior cingulate); worse motor performance and higher burden of emotional symptoms; increased activation associated with worse emotional state; suggests a compensatory recruitment of the cognitive network to maintain performance.
Fahim et al.^15^	Germany	Cross-sectional	Investigate microstructural changes in deep gray matter nuclei after COVID-19	EG (post-COVID): n=69 (hospitalized n=26; mild n=43); ~51 yrs; 15M/11F. Mild home-recovered subgroup: n=43; 42 yrs (median); 18M/25F; ~5 months post-infection; unvaccinated. CG (seronegative, unvaccinated): n=76; ~44.5 yrs; 39M/37F; age/sex comparable.	Technique: 3T quantitative T1 MRI (qT1). Measures: qT1 values and subcortical nucleus volumes (striatum, thalamus, hippocampus); clinical scales.	No differences in the overall group compared with the CG; in participants aged ≥40 years who had been previously hospitalized, increased qT1 was observed in the caudate, putamen, nucleus accumbens, and hippocampus; alterations were independent of cognitive performance or disease duration; olfactory loss was associated with changes in the caudate; suggesting subcortical microstructural alterations related to severity and age.
Du et al.^31^	China	Prospective longitudinal	Investigate brain structural changes and neuropsychiatric symptoms after Omicron infection	EG (post-COVID - Omicron): n = 61; 43.1 ± 9.9 years; all male; fever subgroup n = 36. CG: no independent CG; comparisons were within-subject (pre- vs. post-Omicron) and between febrile vs. non-febrile participants.	Technique: 3T MRI. Measures: Cortical thickness, sulcal depth, subcortical volume (hippocampus); emotional scales and cognitive tests.	Increased anxiety and worse emotional regulation after infection; reduced cortical thickness in occipital regions and the precuneus; reduced right hippocampal volume; structural alterations more evident in individuals with fever; cortical thinning associated with higher anxiety; suggesting early brain structural impact even in mild Omicron infections.
Capelli et al.^18^	Italy	Cross-sectional	Investigate alterations in the olfactory bulb and olfactory tract in patients with COVID-19 and neurological symptoms	EG (post-COVID with neurological symptoms): n = 196; ~53 years; 116F/80M; olfactory dysfunction subgroup n = 78; other neurological symptoms (ND) subgroup n = 118; ~56 years. CG (healthy controls): n = 39; ~55 years; 19F/20M.	Technique: 3T MRI. Measures: Olfactory bulb volume; signal intensity in the olfactory tract.	The EG showed reduced olfactory bulb volume compared with the CG; atrophy was independent of age; no differences were observed between patients with or without isolated olfactory dysfunction; no correlation with time since infection; no consistent differences in the olfactory tract; suggesting persistent structural damage of the olfactory bulb after COVID-19.
Dadsena et al.^40^	Germany	Prospective longitudinal	Investigate the evolution of brain changes and neuropsychiatric symptoms up to 2 years after COVID-19	EG (post-COVID with persistent symptoms): n = 79; 46.4 ± 11.3 years; 48F/31M. CG (healthy controls): n = 21; 40.6 ± 14.5 years; 8F/13M; age- and sex-matched.	Technique: 3T MRI (structural + fMRI). Measures: Gray matter volume; functional connectivity; cognitive, fatigue, and sleep scales.	The EG showed reduced cortical volume in the early phase (~7 months); structural reorganization over time (regional gains and losses); functional connectivity alterations in multiple regions; partial clinical improvement (fatigue and cognition), with persistence of brain alterations; an initial association between fatigue and brainstem, not maintained at follow-up; suggesting compensatory reorganization with dissociation between symptoms and imaging findings in the long term.
Han et al.^45^	China	Prospective longitudinal	Investigate cortical changes and their relationship with fatigue after mild COVID-19	EG (post-mild COVID): n = 29; 34.3 ± 6.6 years; 17F/12M. CG (healthy controls): n = 24; 31.4 ± 7.3 years; 13F/11M.	Technique: fNIRS + TMS. Measures: Cortical activation (PFC, M1); corticospinal excitability; fatigue and functional scales.	The EG showed reduced prefrontal and motor cortex activation at 1 month; reduced cortical excitability (intracortical inhibition/facilitation); high initial fatigue with improvement over time; lower PFC activation associated with greater fatigue; recovery of M1 excitability associated with fatigue improvement; reinfection associated with worsening of cortical parameters and fatigue; suggesting a neurophysiological basis of fatigue with partial recovery dependent on cortical excitability.
Germann et al.^33^	United Kingdom	Cross-sectional	Assess neurophysiological dysfunction in persistent post-COVID fatigue (cortical excitability, peripheral fatigue, autonomic function, and reaction time)	EG (post-COVID fatigue - pCF): n = 145; 48.2 ± 9.8 years; 108F/37M; non-severe infection (no COVID-19 hospitalization). Subgroup pCF <200 days: n = 44; 46.7 ± 9.1 years; 31F/13M. Subgroup pCF >200 days: n = 101; 48.8 ± 10.1 years; 77F/24M. CG (healthy controls): n = 52; 46.6 ± 9.4 years; 37F/15M; no fatigue, some with prior COVID-19 but recovered.	Technique: TMS + peripheral electrophysiology + autonomic and reaction-time tests. Measures: Cortical excitability (RMT, AMT, SICI, ICF, CSP); peripheral fatigue/central activation; reaction time; HRV; SpO₂; FIS.	Compared with the CG, the total EG (pCF) showed reduced ICF, increased cortical excitability (↓RMT/AMT), greater peripheral fatigue, reduced central activation, longer reaction times, and dysautonomia (↑HR, ↓HRV, ↓SpO₂) (p < 0.05); the >200-day subgroup showed worse fatigue (FIS), higher cortical excitability, greater peripheral fatigue, greater central deficit, and slower reaction times (p < 0.05); autonomic parameters did not differ between subgroups.
Gangemi et al.^25^	Italy	Longitudinal observational	Investigate changes in the cortical activity (P300 and EEG rhythms) in patients with post-COVID cognitive fog	EG1 (cognitive fog without anosmia): n = 10; mean age 59.8 ± 12.9 years; 50% female. EG2 (cognitive fog with anosmia): n = 10; same age range. CG (healthy controls): n = 20; mean age 59.8 ± 12.9 years; 50% female.	Technique: EEG (auditory oddball paradigm). Measures: P300 (latency, amplitude); spectral power (alpha, beta, theta bands).	No differences were found between subgroups with vs. without anosmia; compared with the CG, the EG showed increased P300 latency (p < 0.01), reduced alpha and beta power (p < 0.01), and increased theta power (p < 0.01); alterations remained stable between 1 and 8 months.
Appelt et al.^26^	Brazil	Prospective longitudinal	Examine changes in brain electrical activity and cognitive function after mild to moderate COVID-19 over a 12-month period.	EG (mild to moderate COVID-19): n = 53; ~42.3 years; 69.8% female; assessments at 0-3, 3-6, and 6-12 months; non-hospitalized. CG: n = 30; ~37.9 years; 73.3% female.	Technique: EEG (resting state and TMT A/B tasks). Measures: Hjorth parameters (activity, mobility, complexity); cognition (MoCA, TMT A/B, Digit Span).	The EG showed reduced activity in Fz-F4 (resting state) and F3-F7 (TMT B) and reduced complexity in F3-F7 (p < 0.05); worse cognitive performance (increased TMT A completion time) at 6-12 months compared with the CG (p = 0.04); correlations between EEG parameters and cognitive performance.
Babiloni et al.^24^	Italy	Cross-sectional	Assess resting-state EEG rhythms in post-COVID patients with ‘brain fog’ without objective cognitive deficits	EG (brain fog): n = 36; 59.8 ± 1.0 years; 21F/15M; ~1-year post-infection; previously hospitalized; no cognitive or psychiatric deficits. CG (healthy controls): n = 15; 59.1 ± 1.7 years; 8F/7M.	Technique: High-density EEG (64 channels, resting state). Measures: Current density by frequency band (delta-gamma) in cortical ROIs (eLORETA).	The EG showed reduced alpha activity (alpha2/alpha3) in the occipital, parietal, and temporal regions (p < 0.001); a more pronounced reduction in patients with greater fatigue (p = 0.03); no differences in other frequency bands and no correlation with cognitive performance.
Casula et al.^41^	Italy	Cross-sectional (case-control)	Assess the TMS-evoked cortical oscillatory activity in patients with post-COVID fatigue	EG (post-COVID fatigue): n = 16; 40.8 ± 9.3 years; 9F/7M; ~196 days post-infection; non-hospitalized. CG (healthy controls): n = 12; 43.3 ± 10.9 years; 9F/3M.	Technique: TMS-EEG (stimulation of M1 and SMA). Measures: TEPs; oscillatory activity (theta, alpha, beta, gamma).	The EG showed reduced the TMS-evoked beta activity in the SMA (p < 0.05), correlated with greater fatigue (r = -0.596); reduced beta and gamma activity after M1 stimulation in frontoparietal regions (p = 0.025); no differences in the TEPs.
Manganotti et al.^27^	Italy	Cross-sectional	Assess cortical excitatory and inhibitory circuits in patients with long COVID and brain fog	EG (Long COVID - cognitive complaints): n = 18; 50 ± 11 years; 12F/6M. CG (healthy controls): n = 16; 50 ± 7 years; 10F/6M.	Technique: TMS (M1, FDI EMG). Measures: Cortical excitability (RMT, MEP); intracortical inhibition/facilitation (SICI, ICF, LICI, SAI).	The EG showed reduced LICI (GABAa) (p = 0.003) and reduced ICF (glutamatergic) (p < 0.001); no differences in RMT, MEP, SICI **(GABAb),** or SAI (cholinergic); worse cognitive performance (MoCA and executive functions) (p < 0.05).
Ortelli et al.^28^	Italy/Austria	Cross-sectional	Assess the resting-state brain activity (source EEG) in patients with post-COVID syndrome with fatigue and brain fog	EG (cognitive complaints and fatigue): n = 18; 41.1 ± 9.1 years; 10F/8M. CG (healthy controls): n = 18; 48.3 ± 18.1 years; 8F/10M.	Technique: High-density EEG (64 channels, eyes open/eyes closed resting state). Measures: Current density by frequency band (delta-gamma) using eLORETA.	The EG showed a reduced delta activity in the frontoparietal regions and left temporal region (p < 0.01) in the eyes-open condition; delta activity was correlated with greater perceived cognitive difficulty; no differences were observed in other frequency bands or in the eyes-closed condition.
Ortelli et al.^48^	Italy	Cross-sectional	“Assess motor cortex physiology and executive functions in post-COVID patients with fatigue and brain fog	EG (mild post-COVID with fatigue and/or cognitive difficulties): n = 67; 49.7 ± 13.3 years; 50F/17M. CG (healthy controls): n = 22; 46.4 ± 14.2 years; 11F/11M.	Technique: TMS (M1) + neuropsychological assessment. Measures: Cortical excitability (RMT, MEP, SP); intracortical inhibition/facilitation (SICI, ICF, LICI, SAI); cognition (MoCA, FAB, attention).	The EG showed higher RMT, lower MEP, and increased SP; reduced LICI (GABAb) and SAI (cholinergic), with no changes in SICI/ICF; worse cognitive performance (executive functions and attention) and longer reaction time.
Nada et al.^39^	Egypt/Saudi Arabia	Retrospective	Describe neuroimaging patterns and their association with clinical outcomes in children with post-COVID neurological symptoms	EG (children with COVID-19): n = 95; mean age 8.2 ± 4.2 years; 53.7% female. CG: not available / not included.	Technique: 1.5T MRI. Measures: Brain and spinal cord structural abnormalities. Measures included patterns of white matter involvement, ischemia, micro- and macro-hemorrhage, diffusion restriction, myelitis, cranial nerve and cauda equina enhancement, acute necrotizing encephalopathy, posterior reversible encephalopathy syndrome, and follow-up findings (resolution, encephalomalacia, myelomalacia)	White matter lesions were the most frequent finding; ischemia and hemorrhage were associated with worse outcomes; some findings resolved on follow-up, although residual sequelae were observed in certain cases; suggesting a heterogeneous spectrum of pediatric post-COVID neurological involvement.
Chen et al.^38^	Taiwan	Cross-sectional	Investigate the neuropsychiatric symptoms and visual perception in children and adolescents with long COVID and their association with brain volume	EG (children with Long COVID): n = 60; 9.9 ± 2.5 years; 43% female; mild disease; assessment 5-8 months post-infection; subgroups: severe symptoms (SN, n = 31) vs mild/absent symptoms (MN, n = 29). CG (healthy controls): age-matched healthy children.	Technique: 3T MRI (T1-MPRAGE). Measures: Gray matter volume (FreeSurfer); visual perception (TVPS-4); neuropsychiatric scale (wNPSS).	Compared with the mild symptom group (MN), the severe symptom group (SN) showed increased left amygdala volume (p = 0.048) and increased left temporal regions; symptom severity correlated with increased temporal gray matter volume; visual perception deficits (especially sequential memory) compared with the CG (p = 0.017); right hippocampal volume correlated with worse visual performance.
Wang et al.^37^	China	Retrospective (case-control)	Investigate the structural brain changes and covariance network alterations in children after COVID-19	EG (post-COVID): n = 26 (longitudinal subsample n = 11); 10.5 ± 3.7 years; mild to moderate infection; assessment ~4 months post-infection. CG (healthy controls): n = 26; 10.9 ± 4.1 years; age- and sex-matched.	Technique: 3T MRI. Measures: Cortical volume, cortical thickness, sulcal depth; network metrics (global efficiency, small-worldness, path length).	The EG showed reduced cortical volume in the frontal, temporal regions, hippocampus, and cingulate cortex; additional longitudinal reductions in motor and cingulate regions; alterations in cortical thickness and sulcal morphology in the superior temporal gyrus; decreased global efficiency and increased network reorganization (higher small-worldness, increased characteristic path length); indicating reduced global integration and structural brain reorganization; suggesting persistence of alterations with partial recovery in some regions.

**Legend: AD:** axial diffusivity; **ALFF:** low-frequency amplitude fluctuation; **ALPS:** perivascular lymphatic system analysis index; **AMT:** active motor threshold; **ASL:** arterial spin labeling; **BOLD:** blood oxygenation level dependent; **CBF:** cerebral blood flow; **CG:** control group; **CSF:** cerebrospinal fluid; **CT:** computed tomography; **DAN:** dorsal attention network; **DKI:** diffusion kurtosis imaging; **DMN:** default mode network; **DSST:** Digit Symbol Substitution Test; **DTI:** diffusion tensor imaging; **EEG:** electroencephalography; **EG:** experimental group; **eLORETA:** exact low-resolution electromagnetic brain tomography; **F: female**; **FA:** fractional anisotropy; **fALFF:** fractional ALFF; **FC:** functional connectivity; **FDI:** first dorsal interosseous; **FIS:** Fatigue Impact Scale; **fMRI:** functional magnetic resonance imaging; **fNIRS:** functional near-infrared spectroscopy; **GABAa:** gamma-aminobutyric acid type A receptor; **GABAb:** gamma-aminobutyric acid type B receptor; **GFAP:** glial fibrillary acidic protein; **GMV:** gray matter volume; **HR:** heart rate; **HRV:** heart rate variability; **ICF:** intracortical facilitation; **ICU:** intensive care unit; **ISOVF:** isotropic volume fraction; **LICI:** long-interval intracortical inhibition; **M:** male; **M1:** primary motor cortex; **MD:** mean diffusivity; **MEP:** motor evoked potential; **MK:** mean kurtosis; **MN:** mild/absent symptoms; **MoCA:** Montreal Cognitive Assessment; **mOFC:** medial orbitofrontal cortex; **MRI:** magnetic resonance imaging; **NfL:** neurofilament light chain; **NODDI:** neurite orientation density and dispersion imaging; **OB:** olfactory bulb; **OD:** orientational diffusivity; **ODI:** orientation dispersion index; **OT:** olfactory tract; **PACS:** acute post-COVID syndrome; **PASC:** post-acute sequelae of COVID-19; **pCF:** post-COVID fatigue; **PET:** positron emission tomography; **PFC:** prefrontal cortex; **qT1:** quantitative T1 mapping; **rCBF:** regional cerebral blood flow; **RD:** radial diffusivity; **RMT:** resting motor threshold; **ROIs:** regions of interest; **SAI:** short-latency afferent inhibition; **SICI:** short-interval intracortical inhibition; **SMA:** supplementary motor area; **SN:** severe symptoms; **SP:** silent period; **SPECT:** single-photon emission computed tomography; **TBSS:** tract-based spatial statistics; **TEP:** TMS-evoked potential; **TMS:** transcranial magnetic stimulation; **TMS-EEG:** TMS combined with EEG; **TMT:** Trail Making Test; **TVPS-4:** Test of Visual Perceptual Skills, 4th edition; **VBM:** voxel-based morphometry; **Vic:** intracellular water fraction; **Viso:** isotropic compartment fraction; **WMH:** white matter hyperintensities; **wNPSS:** Weighted Neuropsychiatric Severity Scale.

### Structural Neuroimaging Findings

Reduced cortical thickness was observed in the limbic and olfactory regions^11^ ^-^ ^13^, along with a volumetric reduction of the hippocampus^12^ ^,^ ^14^ ^,^ ^15^, and the olfactory bulb^16^ ^-^ ^19^. Severe cases presented microvascular abnormalities in the corpus callosum and juxtacortical areas^20^. In patients with mild to moderate COVID-19, however, significant structural changes may be absent or undetectable by conventional MRI^21^. 

### Functional and Neurophysiological Findings

During working memory tasks, compensatory activation was observed in the superior frontal gyrus^22^. In the monetary incentive task, patients showed neural hypoarousal with a temporal delay in frontal activation^23^, reduced posterior alpha activity^24^, increased P300 latency and reduced beta power^25^, reduced signal complexity^26^, GABAergic dysfunction (reduced long-interval intracortical inhibition) and glutamatergic dysfunction (reduced intracortical facilitation) with elevated resting motor threshold and reduced motor evoked potential^27^ ^,^ ^28^, and reduced activation of the prefrontal and orbitofrontal cortex during walking^29^.

### Blood Biomarkers

Elevated neurofilament light chain (NfL), glial fibrillary acidic protein (GFAP), and sCD163 in the acute phase in severe cases showed a partial reduction after three months^30^; GFAP and proteomic alterations (TREM2, ARHGAP1, CASQ2) were independent predictors of cognitive impairment^13^.

### Longitudinal Studies

Longitudinal studies showed a partial recovery of gray matter volume after two years^31^ and partial recovery of cerebral perfusion over time, despite persistent structural changes in the white matter tracts and subcortical nuclei^32^. Persistence of white matter anomalies^26^ ^,^ ^31^ ^,^ ^33^ ^,^ ^34^, increased axial diffusivity^35^ ^,^ ^36^, and reduced axonal density in tracts such as the corona radiata, corpus callosum, and superior longitudinal fasciculus^34^ ^,^ ^36^ persisted for up to two years.

### Pediatric Population

In the pediatric population, cortical volume reduction was observed in the middle cingulate gyrus and left postcentral gyrus, with partial recovery^37^. An increase in the volume of the amygdala and middle temporal gyri was reported in children with severe symptoms^38^. Non-confluent white matter lesions were also observed, with complete resolution in most cases^39^.

### Clinical Correlations

Fatigue was associated with 35 regions showing altered connectivity^40^, changes in EEG, rs-fMRI, ASL, texture, and neurotransmission^12^ ^,^ ^23^; reduction of beta oscillations in the supplementary motor area^41^; and microstructural alterations in the putamen, pallidum, thalamus, and brainstem^42^. Longitudinal analyses identified a distinct thalamo-cortical correlation between fatigued and non-fatigued patients^43^, and right occipital hypoperfusion correlated with physical fatigue^44^. Additionally, reduced prefrontal and motor cortex activation with impaired intracortical excitability was reported in the first month post-COVID, with a recovery of cortical function paralleling fatigue improvement and reinfection exacerbating both brain and clinical measures^45^.

Olfactory dysfunction was associated with olfactory bulb reduction and parietal connectivity alterations, with a negative correlation between the olfactory performance and clustering coefficient^17^as well as reversible changes in the olfactory structural connectivity that normalize within one year regardless of functional recovery^46^.

Post-acute cognitive complaints have been associated with white matter hyperintensity^47^ and reduced delta activity^48^. Frontoparietal hypometabolism was associated with cognitive deficit^49^. Brain fog has been associated with frontoparietal hypometabolism, increased P300 latency, reduced delta activity^25^ ^,^ ^28^ ^,^ ^49^ ^,^ and reduced limbic cortical thickness as well as with increased default mode network connectivity and reduced dorsal attention network connectivity in patients with brain fog^50^. Correlations were also found between regional blood flow and cognitive scores^51^. Headache was associated with suppression of the Hurst exponent^52^.

Overall, the included studies consistently demonstrate that long COVID is associated with multimodal alterations involving structural neuroimaging, functional brain activity, electrophysiological measures, and circulating biomarkers, which correlate with the most prevalent neurological symptoms. To facilitate the integration of these findings, Figure 1 provides a schematic summary of the principal neurophysiological alterations and their associated clinical correlates.

**FIGURE 1:**
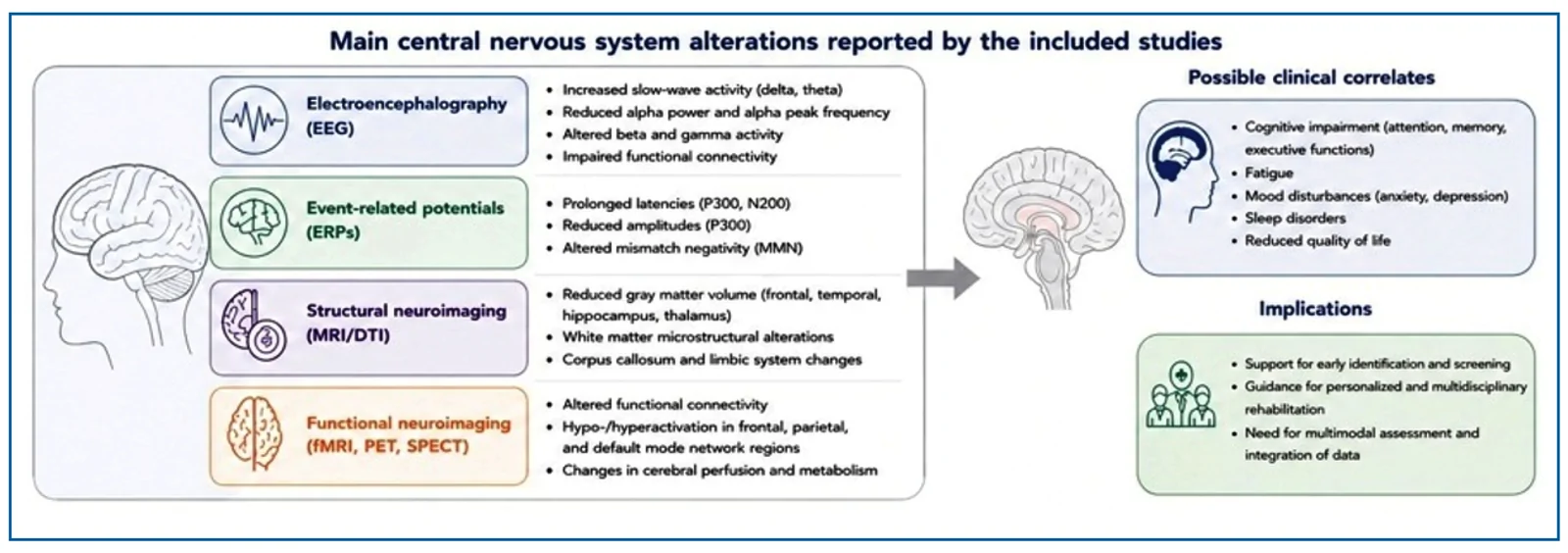
Main Central Nervous System Alterations in Long COVID. Representative diagram summarizing the principal structural, functional, electrophysiological, and biochemical alterations of the central nervous system identified in the studies included in this systematic review. The figure also highlights the main clinical correlates reported across the studies, including fatigue, brain fog, olfactory dysfunction, cognitive impairment, and headache, providing an integrated overview of the current evidence. **Source:** Figure created by the authors with graphic design assistance from ChatGPT (OpenAI, GPT-4o), generated on **7 July 2026**, based on the prompt: *"Scientific infographic summarizing the main central nervous system alterations in Long COVID, organized according to neurophysiological modality and their principal clinical correlates identified in the included studies."*

### Risk of Bias/Methodological Quality

According to the JBI appraisal, 17 studies (40.5%) were rated as high quality (low risk of bias), 19 studies (45.2%) as moderate quality (moderate risk of bias), and 6 studies (14.3%) as low quality (high risk of bias) (Supplementary Table S2). 

## DISCUSSION

This systematic review synthesized evidence from 42 studies investigating CNS alterations after COVID-19 using multimodal neuroimaging, neurophysiological techniques, and blood biomarkers. Overall, the findings support the hypothesis that long COVID manifestations are associated with persistent but heterogeneous brain abnormalities involving structural damage, altered functional connectivity, neurochemical imbalance, and markers of neural injury.

A consistent finding across structural studies was the involvement of limbic, olfactory, and memory-related regions. A reduced cortical thickness in the limbic and olfactory areas was consistently reported^11^ ^-^ ^13^, and hippocampal volume reduction was observed^12^ ^,^ ^14^ ^,^ ^15^. These regions are highly relevant for memory, emotional regulation, and sensory integration, which may help explain frequent complaints such as cognitive dysfunction, anosmia, and mood disturbances in long COVID. Similarly, a reduction in the olfactory bulb volume was found^16^ ^-^ ^19^. This finding reinforces the notion that the olfactory pathway may represent a particularly vulnerable route of injury. It has been further demonstrated that olfactory structural connectivity changes are reversible, normalizing within one year regardless of functional recovery^46^, suggesting that structural reorganization may occur even when clinical symptoms persist. Because olfactory structures maintain close anatomical connections with limbic circuits, damage in these areas may extend beyond smell dysfunction and contribute to broader neurocognitive symptoms.

Nevertheless, the structural abnormalities were not universal. Patients with mild to moderate COVID-19 may show no detectable changes on conventional MRI^21^. This discrepancy likely reflects differences in disease severity, timing of assessment, imaging sensitivity, and sample composition. Conventional MRI may fail to capture the subtle microstructural or metabolic changes that become evident only with advanced techniques such as DTI, functional MRI, PET, or fNIRS. Therefore, the absence of abnormalities on routine imaging should not be interpreted as the absence of CNS involvement.

White matter abnormalities emerged as one of the most persistent findings in longitudinal studies. Persistent white matter alterations have been described^26^ ^,^ ^31^ ^,^ ^33^ ^,^ ^34^, and increased axial diffusivity was observed^34^ ^,^ ^35^. Reduced axonal density in major tracts, including the corona radiata, corpus callosum, and superior longitudinal fasciculus, was also reported^34^ ^,^ ^36^. These pathways are critical for interhemispheric communication, executive processing, and attentional control. Their prolonged disruption may provide a biological substrate for chronic symptoms such as slowed processing speed, attentional deficits, and fatigue. Importantly, while partial gray matter recovery was demonstrated after two years^31^, white matter changes appeared more persistent, suggesting that microstructural connectivity may recover more slowly than macroscopic cortical measures. These longitudinal observations were extended by showing a partial recovery of cerebral perfusion over time, despite persistent structural changes in the white matter tracts and subcortical nuclei^32^. Notably, hypoperfusion was already present in mild cases. The dissociation between reversible vascular abnormalities and persistent structural damage may explain why some symptoms improve while others remain chronic.

Functional and neurophysiological findings suggest that persistent symptoms may result not only from structural injury but also from network dysfunction and altered excitatory-inhibitory balance. During working memory tasks, compensatory superior frontal activation was identified^22^, indicating that the patients may recruit additional neural resources to maintain cognitive performance. Likewise, neural hypoarousal and delayed frontal activation during reward processing were observed^23^, suggesting an inefficiency in motivational and executive circuits. EEG studies reported reduced posterior alpha activity^24^, increased P300 latency and reduced beta power^25^, and lower signal complexity^26^, which are patterns generally associated with impaired information processing and reduced cortical efficiency.

TMS studies further supported neurochemical dysfunction. Reduced long-interval intracortical inhibition and intracortical facilitation, together with elevated resting motor threshold and reduced motor evoked potentials, were reported^27^ ^,^ ^28^. These findings suggest combined GABAergic and glutamatergic dysregulation, possibly linked to neuroinflammation or maladaptive plasticity. A longitudinal perspective in mild COVID-19 revealed reduced prefrontal and motor cortex activation and decreased intracortical inhibition/facilitation within the first month post-infection, which correlated with higher fatigue severity^45^. Over time, the recovery of cortical excitability paralleled fatigue improvement, whereas reinfection led to the worsening of both neurophysiological parameters and fatigue symptoms. Such abnormalities are clinically relevant because they may underlie fatigue, slowed cognition, and motor complaints, while also representing potential therapeutic targets for neuromodulation strategies.

Blood biomarker studies provided complementary evidence of ongoing neural injury and glial activation. Elevated NfL, GFAP, and sCD163 during the acute phase of severe disease were found, with partial reduction after three months^30^. It was further shown that GFAP and proteomic markers such as TREM2, ARHGAP1, and CASQ2 independently predicted cognitive impairment^13^. Together, these findings support the hypothesis that acute neuroaxonal damage and astroglial activation may have lasting clinical consequences. Therefore, biomarkers may become useful tools for identifying patients at higher risk of persistent neurological sequelae.

The symptom-specific analyses were particularly informative. Fatigue, one of the most prevalent long COVID symptoms, was associated with altered connectivity in 35 regions^40^, reduced beta oscillations in the supplementary motor area^41^, microstructural changes in the basal ganglia and brainstem regions^42^, altered thalamo-cortical relationships^43^, and right occipital hypoperfusion^44^. It was further demonstrated that fatigue severity in mild cases is linked to reduced prefrontal cortical activation and impaired intracortical excitability, with clinical and neurophysiological improvement occurring in parallel over time^45^. This convergence suggests that fatigue is not attributable to a single lesion but rather to dysfunction across distributed cortico-subcortical networks involved in arousal, motor initiation, and energy regulation.

Cognitive complaints also demonstrated multi-modal correlates. White matter hyperintensities^47^, reduced delta activity^48^, frontoparietal hypometabolism^49^, altered default mode and dorsal attention networks, increased P300 latency, reduced limbic cortical thickness in brain fog^50^, and correlations between cerebral blood flow and cognitive scores^51^ suggest a disruption of large-scale cognitive control systems. These findings align with patient-reported deficits in concentration, memory, and executive functioning and reinforce that brain fog likely reflects measurable neurobiological dysfunction rather than nonspecific subjective distress.

The pediatric studies reported cortical volume reduction with partial recovery^37^, increased amygdala and middle temporal volumes in children with severe symptoms^38^, and predominantly resolving non-confluent white matter lesions^39^. Although based on only three studies, these results indicate that children may also experience transient or persistent CNS changes after infection. However, developmental brain plasticity, age-related imaging variability, and smaller sample sizes require cautious interpretation.

From a clinical perspective, the present review suggests that persistent neurological symptoms after COVID-19 are biologically plausible and often associated with objective CNS abnormalities. Multidisciplinary evaluation may be warranted in patients with disabling fatigue, cognitive dysfunction, headache, or persistent anosmia, particularly when the symptoms impair daily functioning. The observation that olfactory connectivity normalizes over time provides some prognostic optimism^46^, while the dissociation between perfusion recovery and persistent structural damage highlights that clinical improvement may lag behind certain brain metrics^32^. It has been further suggested that reinfection can worsen both cortical function and fatigue^45^, which has important implications for public health messaging. At the same time, the heterogeneity of findings indicates that no single biomarker or imaging signature currently defines long COVID.

Future research should prioritize prospective multicenter cohorts with standardized definitions, harmonized imaging protocols, repeated longitudinal assessments, and careful adjustment for confounders. The integration of structural imaging, functional connectivity, electrophysiology, and fluid biomarkers may help identify clinically meaningful subtypes and guide targeted rehabilitation or neuromodulatory interventions.

In conclusion, current evidence indicates that long COVID is associated with persistent but variable CNS alterations involving limbic, olfactory, frontoparietal, and white matter networks. While some abnormalities appear to improve over time, others may persist for years. These findings support a neurobiological basis for post-COVID neurological symptoms, although stronger and more standardized evidence is still needed.

## LIMITATIONS

Despite the relevance of these findings, the evidence base has important limitations. The studies were observational, frequently cross-sectional, with modest sample sizes and variable control for confounding factors such as hospitalization severity, premorbid conditions, vaccination status, psychiatric symptoms, and socioeconomic stressors. Considerable heterogeneity was also present regarding the diagnostic criteria for long COVID, symptom duration, imaging protocols, and outcome measures. This heterogeneity prevented formal assessment of inconsistency and publication bias and limits direct comparison across studies. 

## CONCLUSION

The current evidence suggests that the long COVID condition may be associated with a complex pattern of CNS involvement, including structural alterations, connectivity abnormalities, cerebral metabolic changes, cortical excitability dysfunction, and altered oscillatory dynamics.

Rather than a single underlying mechanism, the findings point toward a dysregulation of distributed neural networks, with varying degrees of reorganization and functional compensation, potentially sustaining persistent symptoms even in the absence of overt structural abnormalities.

## DECLARATION OF THE USE OF GENERATIVE AI AND AI-ASSISTED TECHNOLOGIES IN THE WRITING PROCESS

During the preparation of this manuscript, ChatGPT (OpenAI, GPT-4o) was used to assist in language editing, text refinement, and the graphic design of Figure 1. All scientific content, interpretation of the evidence, critical revision, and final approval of the manuscript were performed exclusively by the authors, who assume full responsibility for the accuracy and integrity of the work.

## Data Availability

Research data is available in the body of the article.
