# Supplementary material for: Central Nervous System Alterations in Individuals with Long COVID: A Systematic Review
Source: Rev Soc Bras Med Trop. 2026 Oct 16;59:e0519-2025. doi: 10.1590/0037-8682-0519-2026 (PMC13674201; doi:10.1590/0037-8682-0519-2026)
Supplement: Supplementary file 1 [file 1678-9849-rsbmt-59-e0519-2025-md1.pdf]

Revista da Sociedade Brasileira de Medicina Tropical
Journal of the Brazilian Society of Tropical Medicine

Supplementary Table S1: Complete search strategy for each database.

**PubMed/Medline search strategy**
The following search strategy was applied and adapted to the advanced search mode.
Keywords
("COVID-19"[Mesh] OR "Long COVID"[Mesh] OR "COVID-19"[tiab] OR "SARS-CoV-2"[tiab] OR "long covid"[tiab]) AND ("Central Nervous System"[Mesh] OR "Neurophysiology"[Mesh] OR "EEG"[tiab] OR "electroencephalography"[tiab] OR "TMS"[tiab] OR "transcranial magnetic stimulation"[tiab] OR "cortical excitability"[tiab]) AND (cross-sectional[tiab] OR cohort[tiab] OR longitudinal[tiab] OR observational[tiab] AND "Humans")
**Search strategy on Embase**
The following search strategy was applied and adapted to the advanced search mode.
Keywords
('covid 19'/exp OR 'long covid'/exp) AND (eeg:ti,ab OR tms:ti,ab OR 'cortical excitability':ti,ab OR 'neurophysiology':ti,ab) AND ('observational study'/exp OR 'cohort analysis'/exp OR 'cross-sectional study'/exp)
**Search strategy on Scielo**
The following search strategy was applied and adapted using Portuguese keywords in advanced search mode.
Keywords
(covid longa) OR (covid-19) OR (sars-cov-2) OR (síndrome pós-covid) AND (sistema nervoso central) OR (neurofisiologia) OR (eeg) OR (tms)
**Search strategy on Lilacs**
(tw:("COVID-19" OR "SARS-CoV-2" OR "Síndrome Pós-COVID-19 Aguda" OR "COVID longa")) AND (tw:("Sistema Nervoso Central" OR "Neurofisiologia" OR "EEG" OR "Eletroencefalografia" OR "TMS" OR "Estimulação Magnética Transcraniana")) AND (tw:("estudo transversal" OR "coorte" OR "longitudinal" OR "observacional")) (Humanos)
**Search strategy on Cochrane**
The following search strategy was applied and adapted to the advanced search mode.
Keywords
“covid-19" OR "sars-cov-2" OR "long covid" AND "central nervous system" OR neurophysiology AND “humans”

Caption: Source: own authorship. **Note:** Terms related to study designs (e.g., "cross-sectional," "cohort," "longitudinal," and "observational") were included as free-text terms to maximize search sensitivity rather than applied as methodological filters. No predefined database methodological filters (e.g., "Systematic Review" or "Randomized Controlled Trial") were used to restrict the search.
