# Supplementary material for: Central Nervous System Alterations in Individuals with Long COVID: A Systematic Review
Source: Rev Soc Bras Med Trop. 2026 Oct 16;59:e0519-2025. doi: 10.1590/0037-8682-0519-2026 (PMC13674201; doi:10.1590/0037-8682-0519-2026)
Supplement: Supplementary file 2 [file 1678-9849-rsbmt-59-e0519-2025-md2.pdf]

Supplementary Table S2: Methodological quality and risk of bias of included studies according to Joanna Briggs Institute (JBI) critical appraisal tools.

Study	**Design**	**JBI Tool**	**Overall Quality**	**Main concerns**
Douaud et al., 2022	Longitudinal cohort	JBI Cohort	High	Residual confounding
Esposito et al., 2022	Cross-sectional	JBI Cross-Sectional	Moderate	Sample size
Hartung et al., 2025	Cross-sectional	JBI Cross-Sectional	High	None relevant
Samanci et al., 2025	Cross-sectional	JBI Cross-Sectional	High	None relevant
Seo et al., 2025	Cross-sectional	JBI Cross-Sectional	High	None relevant
Tian et al., 2022	Longitudinal cohort	JBI Cohort	Moderate	Loss to follow-up; exploratory analysis
Scardua-Silva et al., 2024	Cross-sectional	JBI Cross-Sectional	Moderate	DTI-cognition correlations without multiple adjustment
Schmidt et al., 2025	Cross-sectional	JBI Cross-Sectional	High	None relevant
Petersen et al., 2024	Longitudinal cohort	JBI Cohort	Moderate	High loss to follow-up (35%); no analysis of missing data
Huang et al., 2023	Longitudinal cohort	JBI Cohort	Moderate	Small sample size, sex imbalance
Andriuta et al., 2022	Cross-sectional	JBI Cross-Sectional	High	None relevant
Chen et al., 2024	Cross-sectional	JBI Cross-Sectional	Moderate	Self-reported exposure; small sample (fNIRS)
Churchill et al., 2023	Cross-sectional	JBI Cross-Sectional	High	None relevant
Klironomos et al., 2020	Retrospective cohort (descriptive)	JBI Cohort	Low	No control group; descriptive statistics

Huang et al., 2022	Longitudinal cohort	JBI Cohort	Low	Very small sample size, high non-participation rate.
Kandemirli et al., 2021	Cross-sectional	JBI Cross-Sectional	High	None relevant
Haider et al., 2025	Cross-sectional	JBI Cross-Sectional	Low	Large sample (n=112); age- and sex-matched controls
Hosp et al., 2021	Cross-sectional	JBI Cross-Sectional	High	None relevant
Hosp et al., 2022	Cross-sectional	JBI Cross-Sectional	Moderate	Cerebral blood flow differences only in exploratory threshold
Zingaropoli et al., 2023	Longitudinal cohort	JBI Cohort	Moderate	Loss to follow-up of 36%
Leitner et al., 2024	Cross-sectional	JBI Cross-Sectional	Moderate	No adjustments for connectivity confounders.
Bispo et al., 2023	Cross-sectional	JBI Cross-Sectional	High	None relevant
Du et al., 2023	Longitudinal cohort	JBI Cohort	Low	Very small sample (n=16, longitudinal)
Nakase et al., 2026	Cross-sectional	JBI Cross-Sectional	Low	No control group; only internal correlations.
Churchill et al., 2024	Cross-sectional	JBI Cross-Sectional	Moderate	Very small sample
Chang et al., 2023	Cross-sectional	JBI Cross-Sectional	High	None relevant
Fahim et al., 2025	Cross-sectional	JBI Cross-Sectional	High	None relevant
Du et al., 2023	Longitudinal cohort	JBI Cohort	Low	Only men; loss of 28% in the 3rd month
Capelli et al., 2023	Cross-sectional	JBI Cross-Sectional	Moderate	Manual segmentation of the olfactory bulb

Dadsena et al., 2025	Longitudinal cohort	JBI Cohort	High	None relevant
Han et al., 2024	Longitudinal cohort	JBI Cohort	Moderate	Loss of 31% in the 6th month
Germann et al., 2025	Cross-sectional	JBI Cross-Sectional	High	None relevant
Gangemi et al., 2024	Longitudinal cohort	JBI Cohort	Moderate	Database control group
Appelt et al., 2022	Longitudinal cohort	JBI Cohort	Moderate	Loss to follow-up not clearly reported
Babiloni et al., 2024	Cross-sectional	JBI Cross-Sectional	High	None relevant
Casula et al., 2024	Cross-sectional	JBI Cross-Sectional	Moderate	Small sample
Manganotti et al., 2023	Cross-sectional	JBI Cross-Sectional	Moderate	Small sample
Ortelli et al., 2022	Cross-sectional	JBI Cross-Sectional	High	None relevant
Ortelli et al., 2023	Cross-sectional	JBI Cross-Sectional	High	None relevant
Nada et al., 2024	Cross-sectional	JBI Cross-Sectional	Moderate	Retrospective descriptive study
Chen et al., 2025	Cross-sectional	JBI Cross-Sectional	Moderate	No control group for MRI; single-center recruitment
Wang et al., 2025	Cross-sectional	JBI Cross-Sectional	Moderate	Small sample

*Because different JBI tools were used according to study design, the quality ratings were interpreted within each methodological category rather than directly compared across all studies.

Legend: **MRI:** magnetic resonance imaging; **DTI:** diffusion tensor imaging; **fNIRS:** functional near-infrared spectroscopy.
