# Supplementary material for: Central Nervous System Alterations in Individuals with Long COVID: A Systematic Review
Source: Rev Soc Bras Med Trop. 2026 Oct 16;59:e0519-2025. doi: 10.1590/0037-8682-0519-2026 (PMC13674201; doi:10.1590/0037-8682-0519-2026)
Supplement: Supplementary file 3 [file 1678-9849-rsbmt-59-e0519-2025-md3.pdf]

PRISMA 2020 flow diagram for new systematic reviews which included searches of databases and registers only

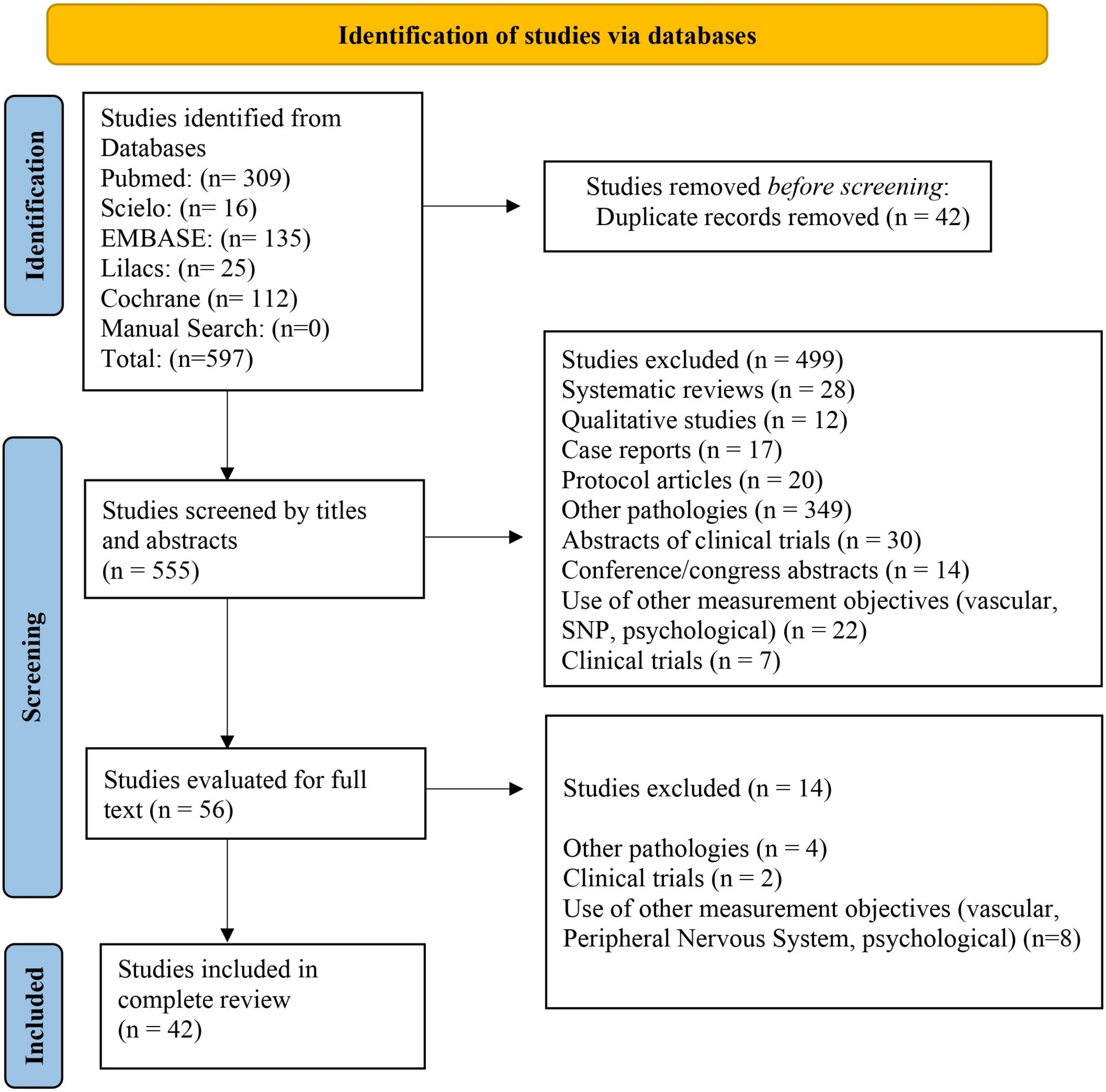

Supplementary Figure S1

Legend: Flow diagram of the study screening and selection process for this systematic review on Central Nervous System alterations in individuals with Long COVID. **Source:** Adapted from Matthew J. Page et al. (2021), *BMJ*, 372: n71. https://doi.org/10.1136/bmj.n71

This work is licensed under a Creative Commons Attribution 4.0 International License (CC BY 4.0). To view a copy of this license, visit: https://creativecommons.org/licenses/by/4.0/
